# Developing a prioritisation framework for patients in need of coronary artery angiography

**DOI:** 10.1186/s12889-021-12088-7

**Published:** 2021-11-03

**Authors:** Leila Doshmangir, Faramarz Pourasghar, Rahim Sharghi, Ramin Rezapour, Vladimir Sergeevich Gordeev

**Affiliations:** 1 Social Determinants of Health Research Center, Tabriz University of Medical Sciences, Tabriz, Iran; 2 Department of Health Policy and Management, School of Management and Medical Informatics, Tabriz University of Medical Sciences, Tabriz, Iran; 3 Student Research Committee, Tabriz University of Medical Sciences, Tabriz, Iran; 4 Institute of Population Health Sciences, Queen Mary University of London, London, UK; 5 Department of Infectious Disease Epidemiology, London School of Hygiene & Tropical Medicine, London, UK

**Keywords:** Coronary artery angiography, Waiting list management, Patient prioritisation, Health policy and services research, Quality of care

## Abstract

**Background:**

Effective waiting list management and comprehensive prioritisation can provide timely delivery of appropriate services to ensure that the patient needs are met and increase equity in the provision of health services. We developed a prioritisation framework for patients in need of coronary artery angiography (CAA).

**Methods:**

We used a multi-methods approach to elicit effective factors that affect CAA patient prioritisation. Qualitative data wase collected using semi-structured interviews with 15 experts. The final set of factors was selected using experts’ consensus through modifed Delphi technique. The framework was finalised during expert panel meetings.

**Results:**

212 effective factors were identified based on the literature review, interviews, and expert panel discussion of them, 37 factors were selected for modifed Delphi study. Following two rounds of Delphi discussions, seven final factors were selected and weighed by ten experts using pair-wise comparisons. The following weights were given: the severity of pain and symptoms (0.22), stress testing (0.18), background diseases (0.15), number of myocardial infarctions (0.15), waiting time (0.10), reduction of economic and social performance (0.12), and special conditions (0.08).

**Conclusion:**

Clinical effective factors were important for CAA prioritisation framework. Using this framework can potentially lead to improved accountability and justice in the health system.

**Supplementary Information:**

The online version contains supplementary material available at 10.1186/s12889-021-12088-7.

## Background

In recent decades, the staggering growth in the number of people on the waiting lists for certain health care services has exacerbated concerns about patients safety, the quality of health care services and risk of injustice, and led to patients’ dissatisfaction [1, 2]. In general, the creation of a waiting list for a health care service can indicate the presence of high and excessive demand, deficient supply, or a lack of effective planning and prioritisation. Regardless of reasons, extended waiting times were previously shown to negatively impact patients health and quality of life [3]. Increased waiting time was also shown to affect the patient’s functional improvement, patient’s performance and recovery after the operation [4, 5].

Different countries have implemented explicit and implicit priority setting processes according to their circumstances. The factors that can determine the order of patients on the waiting list and related waiting time may include non-clinical (e.g., quality of life, patient experiences, duration, distance from residence to hospital) and clinical factors (e.g., sex, pain intensity, complications and disability ([4, 6–8]. In New Zealand and Canada, priority-scoring systems were used to prioritaze the patients, and in Norway, decisions are made in accordance with medical guidelines by allocating ICD10 codes to the medical descriptions [9–12].

Overall, effective prioritisation of patients should be one of the cornerstones of any health care sector and can help to ensure timely service provision that is essential for the improvement of the quality of care. It is also important to observe equity in health care services access and prioritise the provision of health care services in medical centres to patients with greater or more urgent needs. These important indicators should be given special attention in the evaluation of health and treatment departments where clinical and non-clinical factors for prioritisation should be considered simultaneously [13, 14].

Coronary artery disease as the most common type of heart disease and is one of the leading causes of death in various countries [15, 16]. While there are several ways to diagnose coronary artery disease, coronary artery angiography (CAA) remains to be thus far the most definitive way [17]. In Iran, those in need of CAA currently experience long waiting times, sometimes up to five months [18]. Such long waiting times can potentially lead to negative health impacts (e.g., pain, heart function, and deterioration in the quality of life [19, 20]. It was previously suggested that in addition to the clinical factors (based on the severity of the symptoms and the outcome of the exercise test), prioritisation of CAA should also consider social factors [21, 22]. However, in Iran, the current prioritisation process for CAA is based primarily on “first-come, first-serve” basis (or traditional methods). Such prioritisation principle led to observance of a minimum clinical etiquette, reports of injustice, increased the informal patients payments and unnecessary service provision, as it does not account for patient’s needs and urgency of the condition [23–25]. This present study aims to develop a waiting list framework for patients in need of CAA in Iran to ensure timely CAA provision that takes into account both clinical and non-clinical priorities.

## Methods

**Fig. 1:**
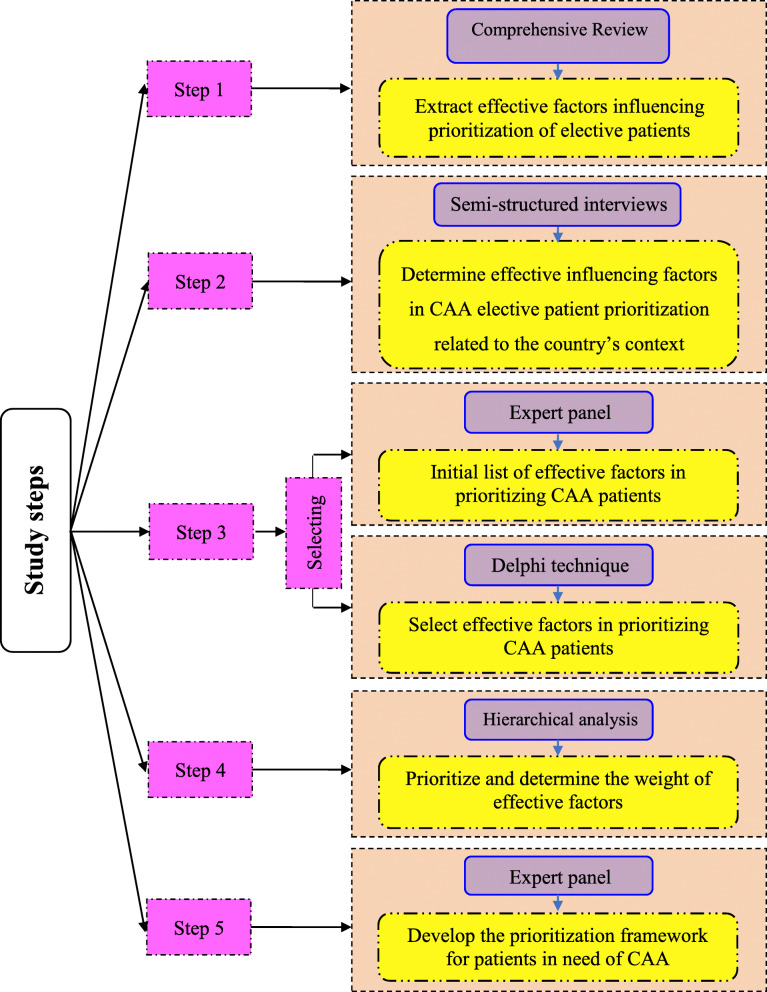
Study Steps

We used a multi-methods approach to elicit effective factors that affected CAA patient prioritisation and conducted our study using five steps (Fig. 1). First, we performed a comprehensive literature review to identify the effective factors influencing the prioritisation of elective patient. Second, we conducted semi-structured interviews to determine effective clinical and non-clinical factors that influence priority setting for CAA patients exclusively related to the country’s context. We interviewed academics, clinical staff and other field experts that are directly involved in the prioritisation of CAA patients. Third, we conducted two sessions with a panel of experts to compile the initial list of effective factors in prioritising CAA patients, which was further narrowed down by using a modifed Delphi technique. All effective factors were judged using two criteria - measurement capability and its importance in prioritising patients in need of CAA. Fourth, we used hierarchical analysis to prioritise the selected effective factors based on their importance in comparison with other factors. Finally, we held two expert panel sessions to formulate the final prioritisation framework for patients in need of CAA based on weights and priority of the effective factors given by experts.

### Step 1: comprehensive review

We conducted a comprehensive literature review to identify the effective clinical and non-clinical factors influencing prioritisation of elective patients. We searched databases (Web of sciences, Scopus, PubMed), using the following terms: waiting list, priority list, priority setting, effecting factors, factor, volunteer patient, elective surgery, non-emergency surgery (Additional file 1). No time limitation was applied, and we included articles that were published in English only. The initial search showed that the number of studies on the prioritisation of cardiac patients and waiting lists related to patients requiring CAA is somewhat limited (less than 83 articles). Hence, we expanded our search strategy to include all studies that reported on the factors influencing the prioritisation of non-emergency patients or the management of waiting lists. We then manually searched for additional references cited in the selected articles.

Given that the selected keywords did not include the words hospital, clinic, and health system, there was a possibility that articles related to waiting list management would include those from other non-health industries (e.g., banking or financial services). Such articles were screened out. Extraction of data from the articles (i.e., methodology, target population, clinical factors and non-clinical factors) was done separately by two researchers (RSh&RR), and in cases of disagreement, the third researcher (LD) helped to reach the consensus. 45 articles (Additional file 5) met our inclusion criteria.we read the articles and through content analysis, we identified 110 clinical and 119 non-clinical factors. After eliminating duplicates and merging similar factors in concept but varying in the formulation, the factors were decreased to 81 clinical and 79 non-clinical factors (Additional file 6).

The knowledge and insights gained at this stage were used to design the interview guide form for semi-structured interviews in Step 2.

### Step 2: semi-structured interviews

In order to elicit the clinical and non-clinical effective factors in prioritising patients who need CAA, we conducted 15 semi-structured interviews with experts, including cardiologists (n = 2) and cardiovascular resident (n = 1), coronary artery angiography specialists (n = 3), angiography nurses (n = 4), faculty members (n = 3) and scheduling experts (n = 2). Participants were selected using targeted sampling with maximum diversity method. The main inclusion criteria for participation were having at least a bachelor’s degree for queuing experts, having at least five years of work experience for cardiovascular specialists, and willingness and consent to participate in research. We got written consent from each participant before participation in the interviews. The interviews were conducted using a semi-structured interview guide, continued until the information saturation was reached, and lasted approximately one hour. To facilitate the interview process, the participants chose the place of the interview. With the conscious consent of the participants, a voice recorder was used. Additionally, notes were taken during most meetings and interviews. Content analysis was used to analyze the data. Data coding was performed by two of the researchers (LD&RR). Six main steps were followed to conduct data analysis: familiarity with the data (data immersion), identification and extraction of primary codes, themes identification (putting primary extracted codes in related themes), reviewing and completing identified themes, naming themes, ensuring the reliability of the extracted codes and themes (reaching an agreement between the two coders by discussing and resolving issues). Data analysis was done by hand.

### Step 3: expert panel and modifed Delphi technique

Excerpts from semi-structured interviews and interview notes were reviewed during two sessions with the panel of experts. The expert panel consisted of three specialists and a cardiovascular resident, four scheduling expert, and two nurses. The initial list of factors influencing the prioritisation of patients with cardiovascular problems was provided to experts in the form of a modifed Delphi technique. The main inclusion criteria for participation in an expert panel were similar to those used in Step 2. The questionnaire was used to perform the modifed Delphi technique, which was performed in two stages. In these questionnaires, each of the factors was scored by experts on two dimensions – measurement capability and its importance in prioritising patients in need of CAA – using a 9-point Likert scale. Factors were selected if their average score was higher than seven. If the median score of the factor ranged from four to seven, the factor was entered into the second stage. All factors with a median score below four were eliminated. An overview of included and eliminated factors in Additional files 2 and 3.

### Step 4: hierarchical analysis

The hierarchical analysis was used to prioritise the selected effective factors based on their importance in comparison with other factors. All factors were subjected to the pair-wise comparison by experts. For each factor in a pair, experts assigned a numerical score that indicated the preference or importance of a factor. Preferential values and corresponding descriptions used for scoring are presented in Additional file 4. Expert Choice software was used to analyse the results. Based on the software output, the priority and weight of the factors were determined.

### Step 5: expert panel

Two expert panel sessions were used to formulate the final prioritisation framework for patients in need of CAA based on weights and priority of effective factors given by experts in Step 4. The main inclusion criteria for participation in an expert panel were similar to those used in previous steps. Experts discussed the framework’s reliability and possible limitations. Each patient was given one of the scales 1 to 10 by the physician according to their circumstances. This process was repeated for all factors. Finally, the score given to each factor was multiplied by the weight gained by the hierarchical analysis factor. In the end, the total score of each patient will be determined the patient’s row in the waiting list.

## Results

### Comprehensive overview

The extracted factors were classified into two categories; clinical and non-clinical factors. The most important clinical factors including pain (n = 24), severity of disease (n = 15) and distress (n = 4) and the most important non-clinical factors including age (n = 15), waiting time (n = 13), ability to work (n = 10), disability (n = 7) and sex (n = 7).

Factors were extracted from studies conducted in 11 countries (Canada (11), New Zealand (8), Spain (7), England (7), Netherlands (5), Finland (2) and Australia (1), Iceland (1), Italy (1), Ireland (1) and Norway (1)). Most studies used a mixed-methods approach (n = 16). Other studies used qualitative methods such as interviews, experts panel and open-ended questionnaires and quantitative methods such as statistical modelling.

#### Semi-structured interview

We interviewed fifteen experts. After implementing and analysing the interviews, the list of factors was limited to 46 clinical and 21 non-clinical factors. After coding all factors, 28 factors were removed due to their generality, non-relevance, and alignment. After eliminating repetitive items, we obtained the final list of 39 effective factors in the prioritisation of CAA patients (Table 1).

**Table 1:** Effective factors in prioritising patients for cardiac angiography/ Extracts from interviews

No.	Effective factors	No.	Effective factors
1	The severity of the pain	21	Social value (apparent value, for example, a rich person)
2	Illness severity (diagnostic group)	22	Priority to be a colleague
3	Disability	23	Electrocardiogram (ECG or EKG^*^)
4	Delay costs	24	History PTCA^*^
5	Number of EF^*^	25	The probability of success after the operation
6	Expected clinical benefit (cost versus efficiency)	26	Underlying diseases such as diabetes, high blood pressure and BMI^*^
7	Number of blocked arteries	27	Being a smoker
8	Diabetes	28	Permission for another operation (priority of another surgery)
9	Exercise test result	29	Recommended patient or hospitalisation
10	Heart pumping power	30	Human capital/ social position
11	Number of previous infarcts	31	Decreased ability of the individual (daily work - job problem)
12	Stress while waiting	32	History of heart valve surgery
13	Family history	33	Risk of death
14	Social issues	34	Special patient conditions (e.g., lack of access in case of urgent need)
15	The importance of the vessel requires intervention (e.g., left main coronary)	35	History of coronary artery intervention
16	Complications of staying on the waiting list	36	Unstable angina
17	The severity of the disease progresses	37	Social dysfunction
18	Possible risk of angiography	38	The importance of the individual for society (e.g., the importance of the surgeon for society)
19	Duration of waiting time	39	Symptoms of psychosis
20	Number of persons under the sponsorship		

Note: EF: Ejection Fraction; ECG/EKG: Electrocardiogram; PTCA: Precutaneous Tranluminal Coronary Angioplasty; BMI: Body Mass Index

#### Expert panel

Based on the expert panel discussion, the set of factors was limited to 21 clinical and 16 non-clinical factors (Additional file 2).

#### Modifed Delphi technique

Thirty-seven factors entered the first stage of modifed Delphi method. In the first stage, after analysing the scores of 15 experts, 17 factors were removed from the study due to low scores. Eight factors achieved a median above 7, and 13 factors advanced to the second stage (Additional files 2 and 3). Considering that the amount of agreement between the opinions in the first and second stages of modifed Delphi was obtained in the remaining 13 factors above 80% [26, 27], so the Delphi ended in the second stage and according to the researcher’s opinions, 11 factors with an agreement rate above 80%, along with eight other factors, were selected as final factors. After two stages of the used delphi technique, the number of factors was limited to six clinical and 13 non-clinical factors. To finalise the list of the affecting factors on prioritisation, we performed an expert panel. Some of the identified factors were merged or removed, reducing the number of factors to seven.

#### Hierarchical analysis

Ten experts that participated in the modifed Delphi study conducted a pair-wise comparison of the remaining seven factors. The resulting weights of factors are ranked and presented in Table 2.

**Table 2:** Final list of factors influencing the prioritisation of angiography candidate patients

No.	Factor	Weights
1	Pain severity and clinical symptoms (type of pain, shortness of breath, weakness, etc.)	0.22
2	Stress test (exercise testing, echo stress, ECG changes)	0.18
3	Underlying diseases (associated) and risk factors (diabetes, high blood pressure, high cholesterol and other diseases)	0.15
4	Number of myocardial infarctions (heart failure rate)	0.15
5	Decreased economic and social performance	0.12
6	Duration of waiting time	0.10
7	Special circumstances (social importance of the individual, being a colleague, etc.)	0.08

#### Expert panel

In order to provide a prioritisation framework for non-emergency patients for angiography, the framework resulting from Step 4 was discussed by the expert panel. Following the discussion, the weights remained unchanged. Within the proposed framework, each factor gained weight. Clinical and non-clinical factors were assigned a score (from 1 to 10). Next, each score was multiplied by its corresponding weight, and the final score per patient was obtained by summing values for all seven factors (Table 3). Patients were then ranked based on their final scores. Based on panel recommendations, patients who get 80 points and higher should be the prioritised and seen within a month, patients with 50 to 80 points would get the second priority and shall be seen within two months, and all other patients get the third priority and shall be seen within three to four months.

**Table 3:** Prioritisation framework for patients requiring coronary artery angiography

	Effective Factors to prioritise patients							
Patient’s name	Pain severity and symptoms	Stress test	Underlying Disease	Number of EF	Duration of waiting time	Decreased economic and social performance	Special circumstances	Total points
	0–22	0–18	0–15	0–15	0–10	0–12	0–8	
Patient A								
Patient B								
Patient C								

Notes: EF: Ejection fraction

## Discussion

We developed a framework for prioritisation of patients in need of CAA based on clinical and non-clinical factors. This framework includes seven effective factors: pain intensity and symptoms, stress test results, underlying diseases, presence of risk factors, the percentage of damage caused by a heart attack, the waiting time, the reduction in economic and social performance and other specific circumstances. Pain intensity and symptoms received the highest weights, while the patient’s special conditions got the lowest weight. We used a multi-methods approach to benefit from the ability to improve the quality of the research process, increase the accuracy and quality of data, generate real information, increase the validity of the study, reveal the various dimensions of the phenomenon under study, strengthen the reliability, validity and comprehensiveness of the study [28].

Our framework accounted for both clinical and non-clinical factors which complement each other. Not surprisingly, the clinical factors that reflect medical urgency received higher weights than non-clinical factors. By including the non-clinical factors were aimed to account for social justice. Previous studies suggest that complex assessment criteria should include a social judgment that it frequently being ignored by the clinical judgment [29, 30]. Nonetheless, others suggested that the number of such non-clinical factors in decision making (i.e., prioritisation) should be limited [31].

Having a better understanding of possible disease complications and related effective factors may help to improve patient selection and reduce mortality during the waiting period. Also, differentiating each criterion and determining the time of occurrence of each of the effective factors in a particular patient can help to regulate the timely receipt of service [32]. In this regard, the results of studies showed that the use of clear and explicit criteria could guarantee better health outcomes and lead to patient satisfaction [33]. Therefore, in the present study, in order to compare patients’ priorities more accurately and fairly when scoring each factor, we considered intensity, extent and effective number in prioritisation, used clear and explicit definitions according to the documents extracted for the criteria. Despite the results of some studies and the attention of some experts to patient factors (e.g., age and sex) in evaluating patients for prioritisation, these factors did not reach the quorum to enter the final framework and were not considered as a prioritisation criterion. Also, in line with these results, other studies have shown that age, ability to pay, treatment costs, education and being under the care of individuals should not have much effect on patient’s prioritisation [34].

The results of the literature review showed that the age factor could not be considered as an important, influential factor for prioritisation [35]. However, some studies suggest that providing medical services to younger patients should be considered, especially for mental health services [36]. Similarly, others suggest that age and sex together should be considered for effective in prioritisation in medical services [37]. We argue that given all aspects of the issue, age and sex can be very important and effective in some diseases but not others. At first, the discussion of sex seems fanatical, but there could be systemic sex-related differences or even discrimination in different cultures and countries. However, with the rise of awareness and the level of cultures, logically, no priority should be given to any sex in identical conditions.

The concept of severity of the disease seems simple, but when it is compared across several patients, it is quite difficult to assess and compare. The severity may be defined as the severity of the pain and the extent of the restrictions or the risk of death. Summarising the results of studies shows that the severity of the disease is the result of the sum of the severity of the factors influencing the decision to treat the disease. As a result, it is not plausible to choose this option as an independent factor [38].

The risk of premature death, as well as the severity of the disease, could be a factor in prioritisation. However, because it is generally expressed and there are several factors affecting death, particularly among angiography candidate, it was not included in the set of final factors. We believe that this factor could be more important in emergency patients and can be more effectively used in the classification of emergency patients [38]. Another influential criterion in prioritising cardiovascular patients is attention to body mass index. Body mass index has been proposed in various studies, two articles related to the heart and one article related to knee replacement. In all three studies, this index is mentioned as an important risk factor and is very important in diseases related to movement organs [39]. Examination of the results of studies showed that high blood pressure could also be considered as a significant factor in prioritisation. The most important complication of high blood pressure is an increased risk of cardiovascular disease. High blood pressure is also a risk factor for heart disease and can be a factor in prioritising angiography candidates [40]. Also, the results of studies showed that having malignant diseases as an underlying disease along with heart disease greatly increases the priority of coronary angiography [41]. Nonetheless, none of these factors reached the quorum by our expert to be included in the final framework.

Among the non-clinical factors, we discussed work relationships, family relationships and informal relationships between people. These factors still lack proper recognition and consideration for patient prioritisation. It should be noted that the perceived value of a person (e.g., based on social status, income or fame) should never be a factor in prioritising because prioritising such factors would mean denying transcendent human values. However, some studies suggest that some medical staff (i.e., surgeons) would consider prioritising hospital staff or personal acquaintances over other patients [35].

The inability to implement the prioritization framework was one of the most important limitations of the present study, which could not be done due to the outbreak of the Coronavirus disease (COVID-19). To overcome this limitation, it is suggested that future researchers design and conducte studies to implement the CAA patient prioritisation framework to evaluate the effectiveness and validity of the framework.

## Conclusion

We propose a comprehensive framework for prioritising patients that require CAA. Our framework accounts for effective factors and risk factors that could lead to receiving timely and quality services according to all clinical and non-clinical factors. Using this tool and replacing it with existing traditional methods of prioritisation can lead to the promotion of accountability and justice in the health system, especially in the provision of medical services, and create a dynamic and secure waiting list. The developed framework can be used in many different circumstances and diseases. We suggest that managers and policymakers implement this framework after modifying and localizing the framework based on local conditions, in order to improve justice and accessibility in organizations and communities. Also we invite colleagues to test this framework, adapt and futher improve it.

## Supplementary Information

**Additional file 1.** Complete search strategy for PubMed databases

**Additional file 2.** Value of factors in first round of Delphi

**Additional file 3.** Agreement rate between the factors entered to the second round of Delphi

**Additional file 4.** Rating method to weighting factors

**Additional file 5.** Characteristics of included studies and the influential factors on prioritization of elective patients

**Additional file 6.** List of influential factors on prioritization of elective patients-extracted from literature review)

## Data Availability

All data generated or analyzed during this study are included in this published article.

## Abbreviation

CAA: Coronary artery angiography

## References

[1] Pope C (1991). Trouble in store: some thoughts on the management of waiting lists. Sociology of Health & Illness.

[2] Cl H, González N, Aguirre U, Blasco J, Elizalde B, Perea E (2010). Can an appropriateness evaluation tool be used to prioritize patients on a waiting list for cataract extraction?. Health Policy.

[3] Street A, Duckett S (1996). Are waiting lists inevitable?. Health policy.

[4] Beanlands RS, Hendry PJ, Masters RG, Woodend K, Ruddy TD (1998). Delay in revascularization is associated with increased mortality rate in patients with severe left ventricular dysfunction and viable myocardium on fluorine 18-fluorodeoxyglucose positron emission tomography imaging. Circulation..

[5] Pitt M, Dutka D, Pagano D, Camici P, Bonser R (2004). The natural history of myocardium awaiting revascularisation in patients with impaired left ventricular function. Eur Heart J.

[6] Rexius H, Brandrup-Wognsen G, Odén A, Jeppsson A (2004). Mortality on the waiting list for coronary artery bypass grafting: incidence and risk factors. Ann Thorac Surg.

[7] Koomen EM, Hutten BA, Kelder JC, Redekop WK, Tijssen JG, Kingma JH (2001). Morbidity and mortality in patients waiting for coronary artery bypass surgery. Eur J Cardiothorac Surg.

[8] Montoya SB, González MS, López SF, Muñoz JD, Gaibar AG, Rodríguez JRE (2014). Study to develop a waiting list prioritization score for varicose vein surgery. Ann Vasc Surg.

[9] Askildsen JE, Kaarbøe O, Holmås TH (2008). Monitoring prioritization in a public health care sector.

[10] Oudhoff JP, Timmermans DR, Rietberg M, Knol DL, van der Wal G (2007). The acceptability of waiting times for elective general surgery and the appropriateness of prioritising patients. BMC Health Serv Res.

[11] Johar M (2014). Are waiting list prioritization guidelines being followed in Australia?. Med Decis Mak.

[12] Kowalewski K, McLennan JD, McGrath PJ (2011). A preliminary investigation of wait times for child and adolescent mental health services in Canada. J Can Acad Child Adolesc Psychiatry.

[13] Wong VW, Lai TY, Lam PT, Lam DS (2005). Prioritization of cataract surgery: visual analogue scale versus scoring system. ANZ J Surg.

[14] Tebé C, Comas M, Adam P, Solans-Domènech M, Allepuz A, Espallargues M (2015). Impact of a priority system on patients in waiting lists for knee arthroplasty. J Eval Clin Pract.

[15] Benjamin EJ, Muntner P, Alonso A, Bittencourt MS, Callaway CW, Carson AP, Chamberlain AM, Chang AR, Cheng S, Das SR, Delling FN, Djousse L, Elkind MSV, Ferguson JF, Fornage M, Jordan LC, Khan SS, Kissela BM, Knutson KL, Kwan TW, Lackland DT, Lewis TT, Lichtman JH, Longenecker CT, Loop MS, Lutsey PL, Martin SS, Matsushita K, Moran AE, Mussolino ME, O’Flaherty M, Pandey A, Perak AM, Rosamond WD, Roth GA, Sampson UKA, Satou GM, Schroeder EB, Shah SH, Spartano NL, Stokes A, Tirschwell DL, Tsao CW, Turakhia MP, VanWagner LB, Wilkins JT, Wong SS, Virani SS, On behalf of the American Heart Association Council on Epidemiology and Prevention Statistics Committee and Stroke Statistics Subcommittee (2019). Heart disease and stroke Statistics-2019 update a report from the American Heart Association. Circulation..

[16] Nesar Hosseini V, Taghipour M, Sharifian R, Hamta A, Feyzi S. Prevalence of coronary artery diseases risk factors in sari-Iran (2005-10). Journal of Gorgan University of Medical Sciences. 2014;15

[17] Afzali S, Masoudi R, Etemadifar S, Moradi M, Moghaddasi J. The effect of progressive muscle relaxation program (PMR) on anxiety of patients undergoing coronary heart angiography. Journal of Shahrekord Uuniversity of Medical Sciences. 2009;11

[18] Aeenparast A, Farzadi F, Maftoon F. Waiting time for specialist consultation in Tehran. Archives of Iranian Medicine (AIM). 2012;15 23199247

[19] Ackerman IN, Bennell KL, Osborne RH (2011). Decline in health-related quality of life reported by more than half of those waiting for joint replacement surgery: a prospective cohort study. BMC Musculoskelet Disord.

[20] Desmeules F, Dionne CE, Belzile E, Bourbonnais R, Frémont P (2010). The burden of wait for knee replacement surgery: effects on pain, function and health-related quality of life at the time of surgery. Rheumatology..

[21] De Bono D, Ravilious B, El-Zoubi I, Dyer T, Podinovskaya Y (1998). A prioritisation system for elective coronary angiography. Heart..

[22] Hadorn DC, Holmes AC (1997). The New Zealand priority criteria project. Part 2: Coronary artery bypass graft surgery. Bmj.

[23] Doshmangir L, Sajadi HS, Ghiasipour M, Aboutorabi A, Gordeev VS (2020). Informal payments for inpatient health care in post-health transformation plan period: evidence from Iran. BMC Public Health.

[24] Rahimi SA, Jamshidi A, Ait-kadi D, Bartolome AR (2014). Applied methods in prioritization of patients in surgery waiting lists. IIE Annual Conference Proceedings.

[25] Priest L (2008). Emergency-room nightmares spur calls for action. Globe and Mail, 3Nov.

[26] Fry M, Burr G (2001). Using the Delphi technique to design a self-reporting triage survey tool. Accid Emerg Nurs.

[27] Landeta J (2006). Current validity of the Delphi method in social sciences. Technol Forecast Soc Chang.

[28] Malina MA, Nørreklit HS, Selto FH (2011). Lessons learned: advantages and disadvantages of mixed method research. Qual Res Account Manag.

[29] Culyer A (1976). Cullis JJJoSP. Some economics of hospital waiting lists in the NHS.

[30] Mariotto A, De Leo D, Buono MD, Favaretti C, Austin P, Naylor CDJTL (1999). Will elderly patients stand aside for younger patients in the queue for cardiac services?. Lancet.

[31] Hador DC, Steering Committee of the Western Canada Waiting List Project (2000). Setting priorities for waiting lists: defining our terms. Cmaj.

[32] Smith DH, Hadorn DC, Steering Committee of the Western Canada Waiting List Project (2002). Lining up for children's mental health services: a tool for prioritizing waiting lists. J Am Acad Child Adolesc Psychiatry.

[33] Testi A, Tanfani E, Valente R, Fato M, Porro I (2009). A web-based system to manage elective waiting lists: efficiency and equity issues. Int J Healthc Technol Manag.

[34] Russell C, Roberts M, Williamson TG, McKercher J, Jolly SE, McNeil J (2003). Clinical categorization for elective surgery in Victoria. ANZ J Surg.

[35] Lewis S, Barer ML, Sanmartin C, Sheps S, Shortt SE, McDonald PW (2000). Ending waiting-list mismanagement: principles and practice. Cmaj.

[36] MacCormick AD, Plank LD, Robinson EM, Parry BR (2002). Prioritizing patients for elective surgery: clinical judgement summarized by a linear analogue scale. ANZ J Surg.

[37] Arnett G, Hadorn DC, Steering Committee of the Western Canada Waiting List Project (2003). Developing priority criteria for hip and knee replacement: results from the Western Canada Waiting List Project. Can J Surg.

[38] Witt J, Scott A, Osborne RH (2009). Designing choice experiments with many attributes. An application to setting priorities for orthopaedic waiting lists. Health Econ.

[39] Dew K, Cumming J, McLeod D, Morgan S, McKinlay E, Dowell A (2005). Explicit rationing of elective services: implementing the New Zealand reforms. Health Policy.

[40] Lack A, Edwards RT (2000). Boland AJJohsr, policy. Weights for waits: lessons from Salisbury.

[41] Martin DK, Walton N (2003). Singer PAJWJoS. Priority setting in surgery: improve the process and share the learning.

