# Supplementary material for: Developing a prioritisation framework for patients in need of coronary artery angiography
Source: BMC Public Health. 2021 Nov 3;21:1997. doi: 10.1186/s12889-021-12088-7 (PMC8565640; doi:10.1186/s12889-021-12088-7)
Supplement: Supplementary file 1 — Additional file 1. Complete search strategy for PubMed databases [file 12889_2021_12088_MOESM1_ESM.docx]

| **Additional file 1:** Complete search strategy for PubMed databases | | |
| --- | --- | --- |
| **Set** | **Strategy** | **Results** |
| #1 | ((waiting list[Title/Abstract]) OR (priority list[Title/Abstract])) OR (priority setting[Title/Abstract]) | 11683 |
| #2 | (effecting factor*[Title/Abstract]) OR (factor*[Title/Abstract]) | 3,491,340 |
| #3 | ((volunteer patient[Title/Abstract]) OR (elective surgery[Title/Abstract])) OR (none-emergency surgery[Title/Abstract]) | 1,258,817 |
| #4 | #1 AND #2 AND #3  ((((waiting list[Title/Abstract]) OR (priority list[Title/Abstract])) OR (priority setting[Title/Abstract])) AND (((volunteer patient[Title/Abstract]) OR (elective surgery[Title/Abstract])) OR (none-emergency surgery[Title/Abstract]))) AND ((effecting factor*[Title/Abstract]) OR (factor*[Title/Abstract])) | 171* |
| *****Filters activated: English | | |

**Developing a prioritization framework for patients in need of Coronary Artery Angiography**

Leila Doshmangir, Faramarz Pourasghar, Rahim Sharghi, Ramin Rezapour, Vladimir Sergeevich Gordeev
