# Supplementary material for: Developing a prioritisation framework for patients in need of coronary artery angiography
Source: BMC Public Health. 2021 Nov 3;21:1997. doi: 10.1186/s12889-021-12088-7 (PMC8565640; doi:10.1186/s12889-021-12088-7)
Supplement: Supplementary file 2 — Additional file 2. Value of factors in first round of Delphi [file 12889_2021_12088_MOESM2_ESM.docx]

**Developing a prioritization framework for patients in need of Coronary Artery Angiography**

Leila Doshmangir, Faramarz Pourasghar, Rahim Sharghi, Ramin Rezapour, Vladimir Sergeevich Gordeev

Additional file 2: Value of factors in first round of Delphi

| **Score of**  **Measurable** | **Score of**  **Importance** | **Factors** | **No** |
| --- | --- | --- | --- |
| 8 | 9 | Severe pain and symptoms | 1 |
| 3 | 7 | Severity of disease | 2 |
| 7 | 5 | Age | 3 |
| 8 | 7.5 | Positive exercise test | 4 |
| 9 | 2 | Marital status | 5 |
| 8 | 7 | Number of myocardial infarctions | 6 |
| 7 | 5 | Intensity of stress while waiting | 7 |
| 7 | 6 | Family history | 8 |
| 8.5 | 2 | Sex | 9 |
| 7 | 3 | The importance of the vessel requires intervention | 10 |
| 7 | 6.5 | Complications of staying on the waiting list | 11 |
| 6.5 | 7 | Probably risk | 12 |
| 7 | 3 | Number of blocked vessels | 13 |
| 7 | 6 | Percentage of clogged arteries | 14 |
| 6 | 5 | The probability of successful action after angiography | 15 |
| 8 | 8 | Accompanying diseases such as diabetes and ... | 16 |
| 8 | 3.5 | Smoking | 17 |
| 6.5 | 5.5 | Allow other surgery | 18 |
| 8 | 8 | ECG changes | 19 |
| 7.5 | 3 | PTCA history | 20 |
| 7 | 3 | CABG history | 21 |
| 7.5 | 6 | History of valve surgery | 22 |
| 6 | 6.5 | Severe disease progression | 23 |
| 6 | 8 | Risk of death | 24 |
| 5 | 3 | Decreased individual ability | 25 |
| 8 | 7 | Waiting time | 26 |
| 5.5 | 3 | Number of dependents | 27 |
| 6 | 6 | Expected profit | 28 |
| 5 | 1 | Social value | 29 |
| 7 | 2 | The priority is to be a partner | 30 |
| 7 | 2.5 | Quality of life | 31 |
| 5.5 | 2.5 | Custom patient | 32 |
| 7 | 8 | Decreased socio-economic performance | 33 |
| 3 | 4 | Social threat | 34 |
| 3.5 | 3 | Drugs and alcohol | 35 |
| 6.5 | 6 | The importance of the individual to society | 36 |
| 8 | 7 | Special condition | 37 |
