# Supplementary material for: Developing a prioritisation framework for patients in need of coronary artery angiography
Source: BMC Public Health. 2021 Nov 3;21:1997. doi: 10.1186/s12889-021-12088-7 (PMC8565640; doi:10.1186/s12889-021-12088-7)
Supplement: Supplementary file 3 — Additional file 3. Agreement rate between the factors entered to the second round of Delphi [file 12889_2021_12088_MOESM3_ESM.docx]

**Developing a prioritization framework for patients in need of Coronary Artery Angiography**

Leila Doshmangir, Faramarz Pourasghar, Rahim Sharghi, Ramin Rezapour, Vladimir Sergeevich Gordeev

Additional file 3: Agreement rate between the factors entered to the second round of Delphi

| **Agreement rate** | **Factors** | **No** |
| --- | --- | --- |
| Lower than 80% | Age | 1 |
| Lower than 80% | Intensity of stress while waiting | 2 |
| Higher than 80 % | Family history | 3 |
| Higher than 80 % | Complications of staying on the waiting list | 4 |
| Higher than 80 % | Probably risk | 5 |
| Higher than 80 % | Probability of successful operation after angiography | 6 |
| Higher than 80 % | Allow other surgery | 7 |
| Higher than 80 % | Valve operation history | 8 |
| Higher than 80 % | Risk of death | 9 |
| Higher than 80 % | Expected profit | 10 |
| Higher than 80 % | The priority is to be a partner | 11 |
| Higher than 80 % | Percentage of clogged arteries | 12 |
| Higher than 80 % | The importance of the individual to society | 13 |
