# Supplementary material for: Developing a prioritisation framework for patients in need of coronary artery angiography
Source: BMC Public Health. 2021 Nov 3;21:1997. doi: 10.1186/s12889-021-12088-7 (PMC8565640; doi:10.1186/s12889-021-12088-7)
Supplement: Supplementary file 4 — Additional file 4. Rating method to weighting factors [file 12889_2021_12088_MOESM4_ESM.docx]

**Developing a prioritisation framework for patients in need of Coronary Artery Angiography**

Leila Doshmangir, Faramarz Pourasghar, Rahim Sharghi, Ramin Rezapour, Vladimir Sergeevich Gordeev

Additional file 4: Rating method to weighting factors

| **Preferential value** | **Compare factor i with j** | **Description** |
| --- | --- | --- |
| **1** | equal importance | factor i is equally important to j or does not take precedence over each other |
| **3** | relatively more important | factor i is slightly more important than j |
| **5** | more importantly | factor i is more important than j |
| **7** | much more important | factor i has a much higher preference than j |
| **9** | quite important | factor i is absolutely more important than j and it is not comparable to j |
| Other values (2,4,6,8) | -- | intermediate values between preferential values. For example, 8 indicates a value greater than 7 but lower than 9. |
