# Supplementary material for: Developing a prioritisation framework for patients in need of coronary artery angiography
Source: BMC Public Health. 2021 Nov 3;21:1997. doi: 10.1186/s12889-021-12088-7 (PMC8565640; doi:10.1186/s12889-021-12088-7)
Supplement: Supplementary file 5 — Additional file 5. Characteristics of included studies and the influential factors on prioritization of elective patients [file 12889_2021_12088_MOESM5_ESM.docx]

**Developing a prioritization framework for patients in need of Coronary Artery Angiography**

Leila Doshmangir, Faramarz Pourasghar, Rahim Sharghi, Ramin Rezapour, Vladimir Sergeevich Gordeev

Additional file 5: characteristics of included studies and the influential factors on prioritization of elective patients

| **Clinical factors** | **Non-clinical factors** | **Study method** | **Country** | **Authors(year)** | **No** |
| --- | --- | --- | --- | --- | --- |
| - Pain - Rate of progress of disease - Ability to influence outcome - Degree of distress | - Disability- - Waiting time - Affecting outcome by delay - Age | Literature review | Canada | Samira Abbasgholizadeh Rahimi1  Et al; 2014(1) | 1 |
| - Gravity of illness - Pain - Probability of recovery | - Age - Restraints on daily activity - Social criteria - Ability to work | Qualitative study:  Literature review | Spain | Cristian tebe et al; 2014(2) | 2 |
| - Clinical manifestations complications- - Varicose vein size | - Quality of life - Working conditions | Quantitative study:  Questionnaire | Spain | Serge bellmunt  Montoya; 2014(3) | 3 |
| - Risk of complications during the waiting period. - Clinical effectiveness of the intervention | - Waiting time - Utilization of healthcare resources and services during the waiting period. - Quality of life | Quantitative study | Spain | Paula  Adam et al; 2010(4) | 4 |
| - Pain - Pain at rest | - Ability to work - Financial difficulties enjoyment of life- | Quantitative study | Australia | Julia Witt and  Et al; 2009(5) | 5 |
| - Pain - Disease severity | - Difficulty in doing activities of daily living - limitations on ability to work - being a caregiver - recovery probability | Mixed study | Spain | Sampietro-Colom L and Et al; 2008(6) | 6 |
| - Pain - BMI - Stiffness joint | - Sex - Age - Functional limitation - Waiting time | Mixed:  Literature review and quantitative study | Spain | Antonio Escobar; et al 2008(7) | 7 |
| - Rate of progress of disease - Severe pain- | - Age - Disability - Dysfunction | Quantitative study | Italy | A. Testi  Et al; 2008(8) | 8 |
| - Physical symptoms - Psychological distress | - Social limitations - Impairments in work | Qualitative study | Netherlands | Jurian  P, et al; 2007(9) | 9 |
| - Moderate pain on motion - Mild pain at rest | - Able to walk 1–5 blocks without significant pain - Limitations on ability - Ability to fulfil their role and independence in society | Qualitative study:  Expert panel | Canada | Carolyn et al; 2005(10) | 10 |
| - | - Age - Waiting time | Quantitative study:  Modeling | England | T Perris and AW Labib; 2004(11) | 11 |
| - Pain on motion - Pain at rest | - Ability to walk - Other functional limitations - Potential for progression of disease - Ability to work - Give care to dependent, live independently - | Qualitative study:  Expert panel | Canada | Gordon Arnett  Et al; 2003(12) | 12 |
| - Pain on motion- - Pain at rest | - Ability to walk - Functional limitation independently live | Qualitative study:  Expert panel | Canada | David Hadom; 2003(13) | 13 |
| - Pain - Age - Clinical evidence compensation pending - Deterioration of disease | - Anticipated benefit - Dependence on others - Disability - Ability to pay - Evidence of cost-effectiveness | Mixed method: Literature review and qualitative study  (questionnaire) | England | Rhiannon Tudor Edwards; 2003(14) | 14 |
| - | - Waiting time - Social factor - Disability factor | Literature review | England | Penelope M. Mullen; 2002(15) | 15 |
| - Best corrected visual acuity   . Glare  ocular -comorbidity   - extent of impairment in visual function | - Other substantial disability - Ability to work | Mixed method:  Literature review and  Quantitative study (questionnaire) | Canada | Kenneth.g  Romanchuk, et al; 2001(16) | 16 |
| - Internalized symptoms - Acute psychosis - Externalized/disruptive behavior | - Danger to self - Danger to others - Age - Social/friendships/community functioning - Family history | Literature review and expert panel | Canada | Derryck H. Smith  Et al; 2002(17) | 17 |
| - Illness severity - Risk of premature death. | - Limitations on activity | Literature review | Canada | Hadorn et al; 2000(18) | 18 |
| - | - Age - Status work - Waiting time | Literature review | Canada | Steven Lewis  Et al; 2000(19) | 19 |
| - Severity of disease | - Age - Waiting time | Quantitative study  Modeling | England | Julie Ratcliffe et al; 2000(20) | 20 |
| - Severe symptoms - Obesity | - Age - Sex - Smokers - Employed - Dependents | Quantitative study:  Modeling | New Zealand | D P de Bono  Et al; 1998(21) | 21 |
| - Prioritizing surgeon | - Age - Sex - Nationality | Quantitative study:  Modeling | New Zealand | Rachel J. Hunter  Et al; 2018(22) | 22 |
| - Ability to work rate of progression future complications- | - Life independently limitations in doing -activities of daily living - Waiting time - Look after others | Literature review and Delphi technique | Spain | Mait solan Domènech et al; 2013(23) | 23 |
| - Clinical disorders | - Difficulty in doing activities of daily living - Social factor | Quantitative study: Modeling | Spain | Ruben, Roman  Et al; 2008(24) | 24 |
| - Severity of condition- | - Expected benefit - Waiting time | Qualitative study | Norway | Jan Erik Askildsen  Et al; 2008(25) | 25 |
| - Severity of disease | - Age - Sex | Quantitative study: questionnaire | Finland | I. Isojoki; 2008(26) | 26 |
| - Pain | - Social limitations - Impairments in work | Questionnaire | Netherlands | Jurriaan P, Oudhoff; Et al; 2007(27) | 27 |
| - Severity of disease - Pain - LV function - Result of stress testing | - Ability to work   Dependents- | Literature review and review article | Netherlands | N W Jackson  Et al; 1999(28) | 28 |
| - Pain - Severity of disease - Probability of recovery | - Disability - Affecting outcome by delay | Review article and interview | New Zealand | Kevin dew; Et al; 2006(29) | 29 |
| - Emotional distress | - Limitations on ability to work | Interview | Netherlands | G.L.M. Hilkhuysen  Et al; 2005(30) | 30 |
| - Physical role bodily pain vitality - Social functioning - Role emotional mental health- | - Social functional | Descriptive study: questionnaire | New Zealand | J-C. Theis; 2004(31) | 31 |
| - Severity of disease risk - Future complications - Psychological | - Quality of life - Socio-political/logistic | Interview | New Zealand | Andrew MacCormick; 2004(32) | 32 |
| - Pain - Frequent pain | - | Literature review and  Expert panel | Canada | T. W. Noseworthy  Et al; 2003(33) | 33 |
| - Left ventricular function - Myocardial infarction - Congenital heart disease history of hypertension - BMI | - | Quantitative study:  Modeling | Iceland | A. Andrew Ray; 2002(34) | 34 |
| - Malignant disease | - Waiting time - Age - Sex | Quantitative study:  Modeling | New Zealand | A Ndrew  D. M  Ac C Ormick; 2002(35) | 35 |
| - Pain | - Ability to work - Sudden threat - Quality of life - Dependents - Disability- - Limitations on ability to work- | Prospective cohort | New Zealand | Sarah  Derrett  Et al; 2002(36) | 36 |
| - Angina - Previous open-heart surgery - Previous PTCA - Positive exercise test - Familial cardiovascular disease - History of hypertension - Diabetes mellitus - History of hypercholesterolemia - Peripheral arterial disease | - Age - Smoking | Prospective cohort | Netherlands | Egbert M. Koomen  Et al; 2000(37) | 37 |
| - Progression of the disease - Pain - Distress | - Dependents - Waiting time | Delphi technique | England | Alastair  Laek; 2000(38) | 38 |
| - Pain on activity - Pain at rest - History of myocardial infarction - ECG changes | - | Review article and  Delphi technique | England | Harry Hemingway;2000(39) | 39 |
| - ECG changes - Positive exercise test | - Waiting time | Prospective cohort | Canada | David A. Alter; 1999(40) | 40 |
| - Severity of disease | - Age - The economic status - Ability to work | Quantitative study: questionnaire | Finland | Ryynänen OP; 1999(41) | 41 |
| - | - Waiting time - Value to society - Drugs - Alcohol consumption - Outcome - Work status | Quantitative study: questionnaire | England | James Neuberger, et al; 1998(42) | 42 |
| - Exercise test - Diabetes mellitus - Previous myocardial infarction | - | Clinical trial | New Zealand | D P de Bono  Etal; 1998(43) | 43 |
| - Pain - Stress tests - Left ventricular function - BMI | - Sex - Age - Smoking | Literature review and quantitative study | northern Irland | F. Kee1 et al; 1996(44) | 44 |
| - Severity of disease exercise test - Left ventricular function | - | A prospective observation | Canada | Jafna L etal; 1996(45) | 45 |

**References:**

1. Rahimi SA, Jamshidi A, Ait-kadi D, Bartolome AR, editors. Applied methods in prioritization of patients in surgery waiting lists. IIE Annual Conference Proceedings; 2014: Institute of Industrial and Systems Engineers (IISE).
2. Tebé C, Comas M, Adam P, Solans‐Domènech M, Allepuz A, Espallargues MJJoeicp. Impact of a priority system on patients in waiting lists for knee arthroplasty. 2015;21(1):91-6.
3. Montoya SB, González MS, López SF, Muñoz JD, Gaibar AG, Rodríguez JREJAovs. Study to develop a waiting list prioritization score for varicose vein surgery. 2014;28(2):306-12.
4. Paula Adam, Catalan Agency for Health Information, Assessment and Quality (CAHIAQ), IBERESPPriority‐setting for elective surgery procedures with waiting lists of the public healthcare system of Catalonia. 2010.
5. Witt J, Scott A, Osborne RHJHe. Designing choice experiments with many attributes. An application to setting priorities for orthopaedic waiting lists. 2009;18(6):681-96.
6. Sampietro-Colom L, Espallargues M, Rodriguez E, Comas M, Alonso J, Castells X, et al. Wide social participation in prioritizing patients on waiting lists for joint replacement: a conjoint analysis. 2008;28(4):554-66.
7. Escobar A, Quintana JM, González M, Bilbao A, Ibañez BJJoeicp. Waiting list management: priority criteria or first‐in first‐out? A case for total joint replacement. 2009;15(4):595-601.
8. Testi A, Tanfani E, Valente R, Ansaldo G, Torre GJJoEiCP. Prioritizing surgical waiting lists. 2008;14(1):59-64.
9. Oudhoff JP, Timmermans D, Knol D, Bijnen A, Van der Wal GJSs, medicine. Prioritising patients on surgical waiting lists: a conjoint analysis study on the priority judgements of patients, surgeons, occupational physicians, and general practitioners. 2007;64(9):1863-75.
10. De Coster C, McMillan S, Brant R, McGurran J, Noseworthy T, practice PCPotWCWLPJJoeic. The Western Canada Waiting List Project: development of a priority referral score for hip and knee arthroplasty. 2007;13(2):192-7.
11. Labib A, Perris T, editors. The prioritisation of organ transplant patient waiting lists: application of fuzzy logic and multiple criteria decision making. 46th Annual Conference of the OR Society; 2004.
12. Arnett G, Hadorn DC, Surgery SCotWCWLPJCJo. Developing priority criteria for hip and knee replacement: results from the Western Canada Waiting List Project. 2003;46(4):290.
13. Hadorn D, research SCotWCWLPJJohs, policy. Setting priorities on waiting lists: point-count systems as linear models. 2003;8(1):48-54.
14. Edwards RT, Boland A, Wilkinson C, Cohen D, Williams JJHP. Clinical and lay preferences for the explicit prioritisation of elective waiting lists: survey evidence from Wales. 2003;63(3):229-37.
15. Mullen PMJEJoOR. Prioritising waiting lists: how and why? 2003;150(1):32-45.
16. Romanchuk KG, Sanmugasunderam S, Hadorn DC, d'Ophtalmologie SCotWCWLPJCJoOJC. Developing cataract surgery priority criteria: results from the Western Canada Waiting List Project. 2002;37(3):145-54.
17. Smith DH, Hadorn DC, Child SCotWCWLPJJotAAo, Psychiatry A. Lining up for children's mental health services: a tool for prioritizing waiting lists. 2002;41(4):367-76.
18. Hador DC, Cmaj SCotWCWLPJ. Setting priorities for waiting lists: defining our terms. 2000;163(7):857-60.
19. Lewis S, Barer ML, Sanmartin C, Sheps S, Shortt SE, McDonald PWJC. Ending waiting-list mismanagement: principles and practice. 2000;162(9):1297-300.
20. Ratcliffe J, Young T, Buxton M, Eldabi T, Paul R, Burroughs A, et al. A simulation modelling approach to evaluating alternative policies for the management of the waiting list for liver transplantation. 2001;4(2):117-24.
21. Kee F, McDonald P, Gaffney BJH. Prioritising the cardiac surgery waiting list: the angina patient's perspective. 1997;77(4):330-2.
22. Hunter RJ, Buckley N, Fitzgerald EL, MacCormick AD, Eglinton TWJAjos. General Surgery Prioritization Tool: a pilot study. 2018;88(12):1279-83.
23. Solans-Domènech M, Adam P, Tebé C, Espallargues MJHp. Developing a universal tool for the prioritization of patients waiting for elective surgery. 2013;113(1-2):118-26.
24. Román R, Comas M, Mar J, Bernal E, Jiménez-Puente A, Gutiérrez-Moreno S, et al. Geographical variations in the benefit of applying a prioritization system for cataract surgery in different regions of Spain. 2008;8(1):32.
25. Askildsen JE, Kaarbøe O, Holmås TH. Monitoring prioritization in a public health care sector. 2008.
26. Isojoki I, Fröjd S, Rantanen P, Laukkanen E, Närhi P, Kaltiala-Heino RJEc, et al. Priority criteria tool for elective specialist level adolescent psychiatric care predicts treatment received. 2008;17(7):397-405.
27. Oudhoff JP, Timmermans DR, Rietberg M, Knol DL, van der Wal GJBHSR. The acceptability of waiting times for elective general surgery and the appropriateness of prioritising patients. 2007;7(1):32.
28. Jackson N, Doogue M, Elliott JJH. Priority points and cardiac events while waiting for coronary bypass surgery. 1999;81(4):367-73.
29. Dew K, Cumming J, McLeod D, Morgan S, McKinlay E, Dowell A, et al. Explicit rationing of elective services: implementing the New Zealand reforms. 2005;74(1):1-12.
30. Hilkhuysen G, Oudhoff J, Rietberg M, Van der Wal G, Timmermans DJPh. Waiting for elective surgery: a qualitative analysis and conceptual framework of the consequences of delay. 2005;119(4):290-3.
31. Theis JJHsmr. Clinical priority criteria in orthopaedics: a validation study using the SF36 quality of life questionnaire. 2004;17(1):59-61.
32. MacCormick A, Macmillan A, Parry BJJohsr, policy. Identification of criteria for the prioritisation of patients for elective general surgery. 2004;9(1):28-33.
33. Noseworthy T, McGurran J, Hadorn D, practice SCotWCWLPJJoeic. Waiting for scheduled services in Canada: development of priority‐setting scoring systems. 2003;9(1):23-31.
34. Ray AA, Buth KJ, Sullivan JA, Johnstone DE, Hirsch GMJC. Waiting for cardiac surgery: results of a risk-stratified queuing process. 2001;104(suppl_1):I-92-I-8.
35. MacCormick AD, Plank LD, Robinson EM, Parry BRJAjos. Prioritizing patients for elective surgery: clinical judgement summarized by a linear analogue scale. 2002;72(9):613-7.
36. Derrett S, Paul C, Herbison P, Williams HJJohsr, policy. Evaluation of explicit prioritisation for elective surgery: a prospective study. 2002;7(1_suppl):14-22.
37. Koomen EM, Hutten BA, Kelder JC, Redekop WK, Tijssen JG, Kingma JHJEjoc-ts. Morbidity and mortality in patients waiting for coronary artery bypass surgery. 2001;19(3):260-5.
38. Lack A, Edwards RT, Boland AJJohsr, policy. Weights for waits: lessons from Salisbury. 2000;5(2):83-8.
39. Hemingway H, Crook AM, Feder G, Dawson JR, Timmis AJTL. Waiting for coronary angiography: is there a clinically ordered queue? 2000;355(9208):985-6.
40. Alter DA, Basinski AS, Cohen EA, Naylor CDJC. Fairness in the coronary angiography queue. 1999;161(7):813-7.
41. Ryynänen O-P, Myllykangas M, Kinnunen J, Takala JJSs, medicine. Attitudes to health care prioritisation methods and criteria among nurses, doctors, politicians and the general public. 1999;49(11):1529-39.
42. Neuberger J, Adams D, MacMaster P, Maidment A, Speed MJB. Assessing priorities for allocation of donor liver grafts: survey of public and clinicians. 1998;317(7152):172-5.
43. De Bono D, Ravilious B, El-Zoubi I, Dyer T, Podinovskaya YJH. A prioritisation system for elective coronary angiography. 1998;79(5):448-53.
44. Kee F, McDonald P, Kirwan J, Patterson C, Love GJQmjotAoP. The stated and tacit impact of demographic and lifestyle factors on prioritization decisions for cardiac surgery. 1997;90(2):117-23.
45. Cox JL, Petrie JF, Pollak PT, Johnstone DEJJotACoC. Managed delay for coronary artery bypass graft surgery: the experience at one Canadian center. 1996;27(6):1365-73.
