# Supplementary material for: Developing a prioritisation framework for patients in need of coronary artery angiography
Source: BMC Public Health. 2021 Nov 3;21:1997. doi: 10.1186/s12889-021-12088-7 (PMC8565640; doi:10.1186/s12889-021-12088-7)
Supplement: Supplementary file 6 — Additional file 6. List of influential factors on prioritization of elective patients-extracted from literature review) [file 12889_2021_12088_MOESM6_ESM.docx]

**Developing a prioritization framework for patients in need of Coronary Artery Angiography**

Leila Doshmangir, Faramarz Pourasghar, Rahim Sharghi, Ramin Rezapour, Vladimir Sergeevich Gordeev

Additional file 6: list of influential factors on prioritization of elective patients-extracted from literature review

| **No** | **Non-clinical factors** | **No** | **Clinical factors** |
| --- | --- | --- | --- |
| 1 | Waiting time | 1 | pain |
| 2 | Delay costs | 2 | degree of distress |
| 3 | Age | 3 | Stress while waiting |
| 4 | Sex | 4 | Rate of progression |
| 5 | Limitations on activity daily | 5 | Severity of disease |
| 6 | Ability to work | 6 | probability of recovery |
| 7 | Quality of life | 7 | Clinical manifestations |
| 8 | Disability | 8 | Complications |
| 9 | Efficiency of resources in waiting time | 9 | Varicose vein size |
| 10 | Impact on quality of life | 10 | Risk of complications during the waiting period |
| 11 | Job | 11 | Clinical effectiveness of the intervention |
| 12 | Walking restrictions | 12 | pain at rest |
| 13 | Dependents | 13 | BMI |
| 14 | Enjoying the life | 14 | Stiffness joint |
| 15 | Limitations on ability to work- | 15 | Pain or dysfunction |
| 16 | Give care to dependent | 16 | Physical symptoms |
| 17 | Physical performance limitations | 17 | Moderate pain on motion |
| 18 | Disability, dependence on others | 18 | Mild pain at rest |
| 19 | Loss of job and ability to work | 19 | Match ability |
| 20 | Social constraints | 20 | Adaptability |
| 21 | Disorder at work | 21 | Sensitization |
| 22 | Functional limitations | 22 | Pain (in movement and rest) |
| 23 | Independents | 23 | Clinical evidence |
| 24 | Threat to society | 24 | Level of distress |
| 25 | Ability to progress | 25 | visual acuity |
| 26 | Taking care of dependents | 26 | Extent of impairment in visual function |
| 27 | Functional limitations other than walking | 27 | Symptoms of psychosis |
| 28 | Ability to walk | 28 | Comorbidities disease |
| 29 | Functional limitations | 29 | Severity |
| 30 | Predicted profit | 30 | risk of premature death |
| 31 | Dependence on others | 31 | Severe symptoms |
| 32 | Inability to perform normal activities | 32 | Obesity |
| 33 | Social factors | 33 | prioritizing surgeon |
| 34 | Use of resources | 34 | Pain at work |
| 35 | Ability to perform | 35 | Probability of progress |
| 36 | Ability to work | 36 | Complications Probability |
| 37 | Ability to live independently | 37 | Visual disorders |
| 38 | Ability to care for dependents | 38 | Number of vessels taken |
| 39 | Danger to yourself | 39 | Percentage of clogging vessel |
| 40 | Danger to others | 40 | Type of blocked vessel |
| 41 | Function or factors affecting the child | 41 | Left ventricular function |
| 42 | Family performance | 42 | Positive exercise test |
| 43 | Destructive behaviors | 43 | Degree of progress |
| 44 | Family history | 44 | Probability of improvement |
| 45 | Restrictions on activities | 45 | Emotional distress |
| 46 | Occupational status | 46 | Physical role |
| 47 | Time has passed | 47 | general health |
| 48 | Smoking | 48 | mental health |
| 49 | Affiliates | 49 | Emotional role |
| 50 | Evidence of cost-effectiveness | 50 | Risk |
| 51 | Nationality | 51 | Psychological factors |
| 52 | Live independently | 52 | Frequent pain |
| 53 | Restrictions on daily activities | 53 | Heart pumping power |
| 54 | The cost of dependents | 54 | Myocardial infarction |
| 55 | Difficulty in daily activities and ability to work | 55 | Congenital heart disease |
| 56 | Social (one person is needed to care for the patient) | 56 | hypertension |
| 57 | Expected profit | 57 | Body mass index |
| 58 | Inability to play a social role | 58 | malignant disease |
| 59 | Work problems | 59 | Coronary artery occlusion |
| 60 | Ability to work | 60 | History of coronary artery intervention |
| 61 | Disability | 61 | Unstable angina |
| 62 | Delayed clinical cost | 62 | Familial cardiovascular disease |
| 63 | Ability | 63 | Diabetes |
| 64 | Activity restrictions (leaving work) | 64 | History of high blood pressure |
| 65 | Social performance | 65 | hypercholesterolemia |
| 66 | Economic factors | 66 | Peripheral arterial disease |
| 67 | A variety of lifestyles | 67 | Heart valve surgery |
| 68 | Socio-political | 68 | Duration of stable angina |
| 69 | Social issues | 69 | Distress |
| 70 | Sudden threat | 70 | History of myocardial infarction |
| 71 | Quality of life | 71 | change ECG |
| 72 | Smoking | 72 | Dyspnea |
| 73 | Inability to depend on others | 73 | Exercise test result |
| 74 | Maximum recommended waiting time | 74 | Cholesterol levels |
| 75 | The economic situation | 75 | NSD myocardial infarction |
| 76 | Occupational status of the patient | 76 | Stress test |
| 77 | The value of the individual to society | 77 | Left ventricular failure |
| 78 | Alcohol consumption | 78 | BMI |
| 79 | Drug use | 79 | Left ventricular pumping power |
|  |  | 80 | Complications of anxiety |
|  |  | 81 | Stress while waiting time |
